# Exploring Associations Between WaSH-Related Health Outcomes and Terrorist Activities in the Sahel: A Scoping Review

**DOI:** 10.3389/phrs.2025.1608490

**Published:** 2025-10-23

**Authors:** Linda Christina Beck, Branwen Nia Owen, Emma Scott, Mirko S. Winkler, Anaïs Galli

**Affiliations:** ^1^ Department of Health Sciences and Technology, ETH Zürich, Zürich, Switzerland; ^2^ Department of Epidemiology and Public Health, Swiss Tropical and Public Health Institute, Allschwil, Switzerland; ^3^ University of Basel, Basel, Switzerland; ^4^ Department of Epidemiology, Biostatistics and Occupational Health, McGill University, Montreal, QC, Canada

**Keywords:** WaSH, conflict, health outcomes, G5 Sahel, WaSH interventions

## Abstract

**Objectives:**

The G5 Sahel countries have faced political instability and terrorist activities for over a decade. With the regional lack of water, sanitation and hygiene (WaSH), there is an increased risk of adverse health outcomes. This scoping review aims to document WaSH-related health outcomes associated with terrorist activities, identify gaps in the humanitarian and political response and propose actionable recommendations to address them.

**Methods:**

We followed the PRISMA standards, including literature from PubMed and Web of Science. Country-specific timeframes for terrorist activities were used.

**Results:**

Data was extracted from 54 out of 2,320 publications on 22 December 2023. While malnutrition and diarrheal diseases were frequently reported as health outcomes - consistent with inadequate WaSH services - the lack of studies directly linking these outcomes to terrorist activities is notable. Only one article explicitly established a direct link between health outcomes and terrorist activities.

**Conclusion:**

The scarcity of studies directly linking terrorist activities to health outcomes reveals a significant research gap and highlight the need for more focused investigations into the health impacts of political violence in the Sahel region.

## Introduction

Ensuring safe water, sanitation and hygiene (WaSH) is critical for preventing disease and safeguarding health and overall wellbeing [1]. In 2019, nearly 3% of all-age global deaths and 8% of deaths among children under the age of 5 years were attributable to inadequate water, lack of proper sanitation and absence of appropriate hygiene [2]. Inadequate WaSH conditions are closely linked to increased transmission of infectious diseases and increased rates of preventable morbidity and mortality. Preventive measures, such as promoting hygiene practices, establishing proper sanitation facilities and providing access to safe drinking water, could prevent nearly two million deaths annually and significantly reduce disability-adjusted live years (DALYs) for affected populations [2]. The Sahel region bears significant health challenges due to inadequate WaSH, with open defecation common, and access to basic hygiene services limited for many [1, 3]. Poor WaSH conditions result in disproportionally high numbers of deaths, whereas preventive measures in this region could avert one in five deaths among children under five [1].

The Sahel has been affected by complex challenges due to terrorist activities, resulting in political instability [4]. Armed groups such as Jama’at Nasr al-Islam wal Muslimin (JNIM), the Islamic State in the Greater Sahara (ISGS) and Boko Haram are among the most active actors, particularly in Mali, Burkina Faso and Niger. They have contributed to widespread insecurity, undermining state authority and disrupting basic services. The resulting violence and insecurity have led to mass displacement in and across the G5 Sahel countries. In 2025, over 3 million Sahelians were internally displaced persons (IDPs) with 66% of the population in Burkina Faso, followed by 15% and 13% of the population in Niger and Mali [5]. IDP camps, suffer from inadequate WaSH infrastructure compounded by high population density, creating conditions that facilitate the rapid spread of diseases [6]. These settlements are often built on marginal land prone to flooding, with limited drainage and poor-quality infrastructure. In addition, most infrastructure focuses on short-term emergency emergency needs. Consequently, sustainable long-term solutions are often lacking.

In response to these challenges, a range of actors and mechanisms currently operate across the Sahel to coordinate and deliver humanitarian assistance. The Sahel Alliance unites donors and development banks to fund projects in food security, governance, and essential services in G5 Sahel countries [7]. The International Organization for Migration (IOM) provides protection, basic services, and community recovery support to populations affected by conflict and displacement through its Central Sahel Crisis Response Plan [8]. The Centre for Humanitarian Dialogue (HD) supports local peacebuilding and mediation, helping address conflicts in fragile zones [9]. Until recently, the United Nations Multidimensional Integrated Stabilization Mission in Mali (MINUSMA) played a central role in supporting peace and protecting civilians, but the mission was formally ended in 2023, following the Malian government’s request [10]. MINUSMA’s withdrawal has left a gap in international stabilization efforts, with humanitarian actors under increased pressure to fill the void.

Despite these efforts, important evidence gaps remain, particularly regarding regional information about WaSH-specific health outcomes in the general population [11]. Additionally, the ongoing security situation often prevents governments from accessing and supporting affected populations, forcing non-governmental organizations (NGOs) to support [12]. Unfortunately, evidence-based guidance for the prevention of WaSH-related health outcomes in protracted conflicts is limited. By reviewing how WaSH services affect health in the Sahel region, this scoping review aims to (i) document the WaSH-related health outcomes associated with the terrorist insurgency, (ii) identify gaps in the humanitarian and political responses to such health outcomes, and (iii) propose actionable recommendations to address them.

## Methods

### Study Area

**FIGURE 1:**
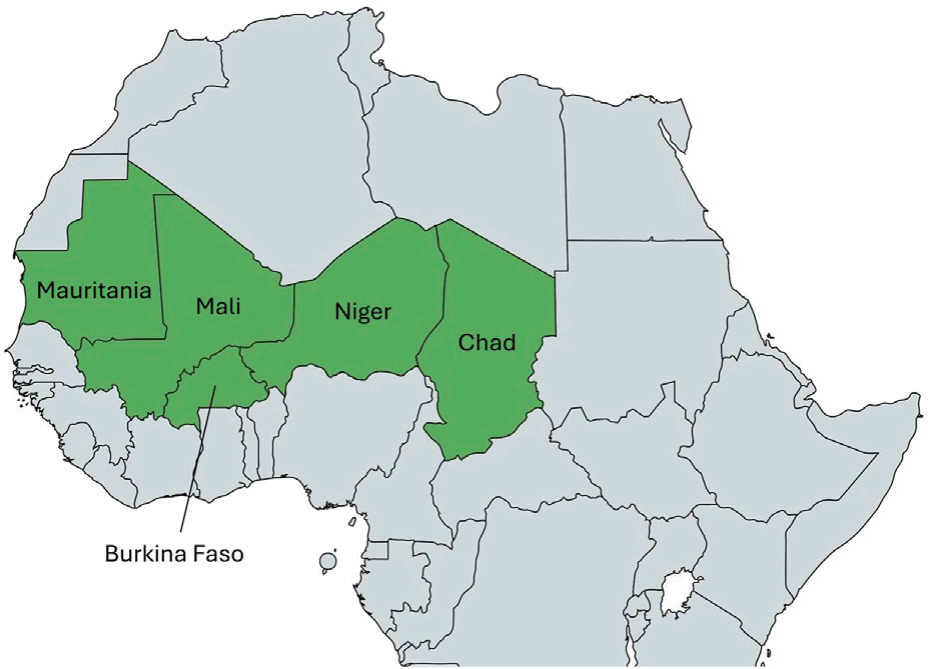
Map of the former G5 Sahel countries [14] (scoping review, Sahel region, 2009–2023).

For this scoping review, we focused on the Sahel region, specifically the former member states of the Group of Five for the Sahel (G5 Sahel) founded in 2014, as illustrated in Figure 1 [13, 14]. This institutional framework originally consisted of the states of Burkina Faso, Chad, Mali, Mauritania and Niger. The alliance’s objective was to unite, strengthening regional security and development through collaborative efforts and to form a peaceful region. Due to recent discrepancies in their political views, Mali, Burkina Faso and Niger left the alliance, whose dissolution has been announced by the end of 2023 [15]. Despite the recent exits of the alliance, we maintained geographic focus in this scoping review on the former five member states because the issues that led to the coalition remain the same.

### Literature Search and Selection Criteria

**FIGURE 2:**
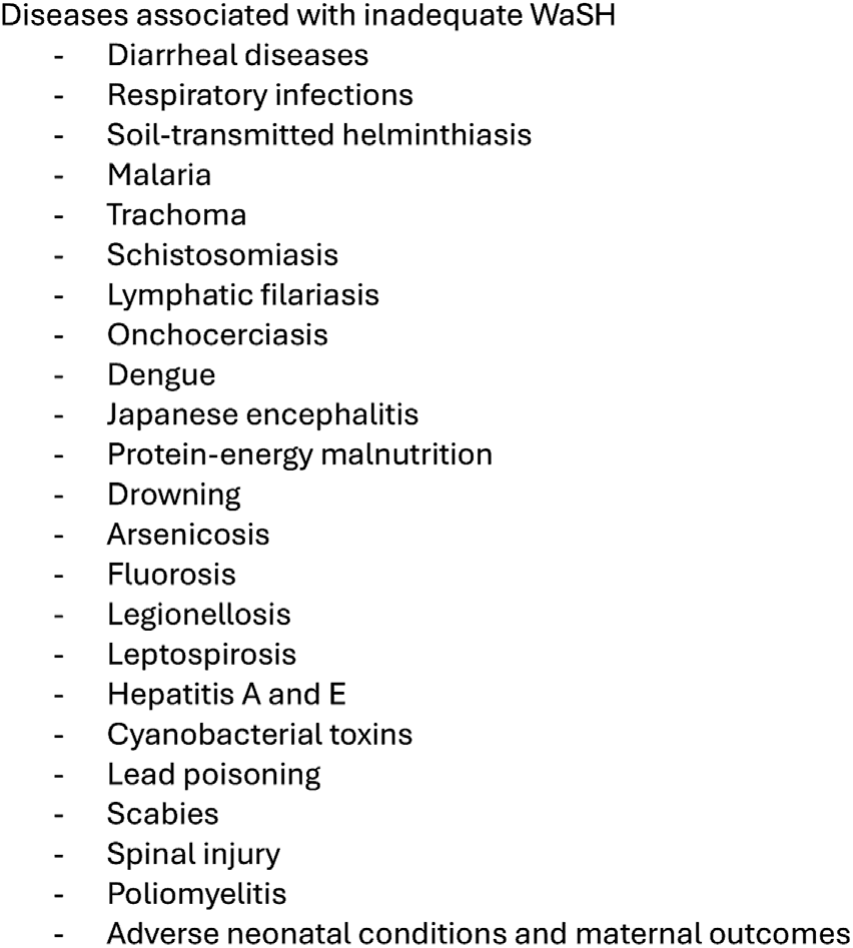
Water, sanitation and hygiene (WaSH) related health outcomes reported by the WHO [1] (scoping review, Sahel region, 2009–2023).

We conducted the review in accordance with Preferred Reporting Items for Systematic reviews and Meta-Analyses (PRISMA) guidelines [16]. We searched PubMed and Web of Science on 22 December 2023, with database-specific syntaxes combining the former G5 countries and terms related to WaSH (see full syntaxes in Supplementary Table S1). To investigate the influence of armed insurgencies, we only included literature starting from the year of onset of terrorist activities in each country. Therefore, literature was searched starting in 2005 for Mauritania, 2012 for Mali and 2015 for Burkina Faso, Chad and Niger [4, 17–19]. To define the diseases resulting from deficient WaSH, we used the list of diseases associated to insufficient WaSH published by the WHO (Figure 2) [1].

We used Covidence [20] to upload and process all records, eliminate duplicates and facilitate the screening process. Inclusion and exclusion criteria (Supplementary Table S2) were applied to titles and abstracts in a double screening process by ES and LB, and discrepancies discussed. The full text of retained articles was then screened by LB. Studies were included if they reported on WaSH-related health outcomes in countries of the former G5 Sahel region, were based on primary data collected after the onset of country-specific insurgencies, were published in English and the full-text was accessible.

The following data were extracted from included articles: authors, year of publication, study period, and investigated country. Additionally, we recorded population characteristics (e.g., age, gender), sample sizes, and study designs. In line with the review objectives, we extracted reported occurrence rates of WaSH-related health outcomes, any explicit or implicit associations of these outcomes with terrorism, preventive measures for WaSH-related health outcomes, and identified gaps in humanitarian assistance. For the purposes of this review, we defined nine categories to classify the obtained health outcomes: (i) malnutrition; (ii) diarrheal diseases; (iii) parasitic infections; (iv) hepatitis; (v) respiratory infections or symptoms; (vi) mosquito-transmitted diseases; (vii) anemia; (viii) leptospirosis; and (ix) scabies. These categories were selected based on their prevalence, relevance, frequent occurrence in the included literature and their documented association with inadequate WaSH conditions [1].

## Results

### Study Selection and Overview

**FIGURE 3:**
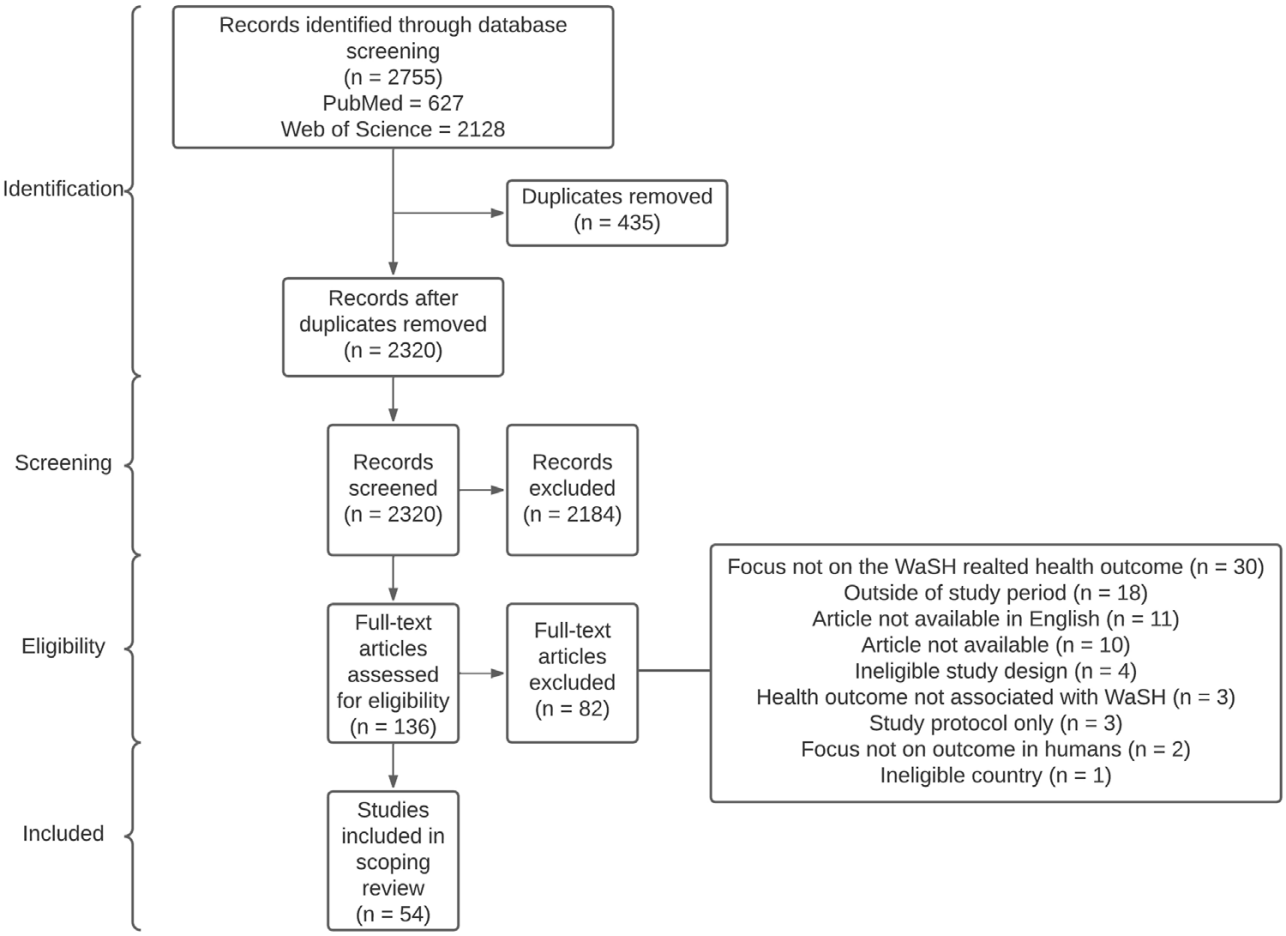
Flowchart representing the literature search and inclusion process based on the PRISMA guidelines (scoping review, Sahel region, 2009–2023).

A total of 2,755 articles were identified, 627 from PubMed and 2,128 from the Web of Science (Figure 3). After removing duplicates, 2,320 unique articles remained for title and abstract screening. During the double-screening process, conflicts arose in approximately 7% (n = 162) of the articles, which were subsequently discussed and resolved. Consequently, 136 articles were retained for full text screening. The most frequent reason for excluding articles was their lack of focus on WaSH-related health outcomes. Other reasons included the non-availability of the full text, a lack of association of the health outcomes to WaSH as defined by the WHO, articles not presented in English, data collection from before terrorist activities or not including any G5 country.

**FIGURE 4:**
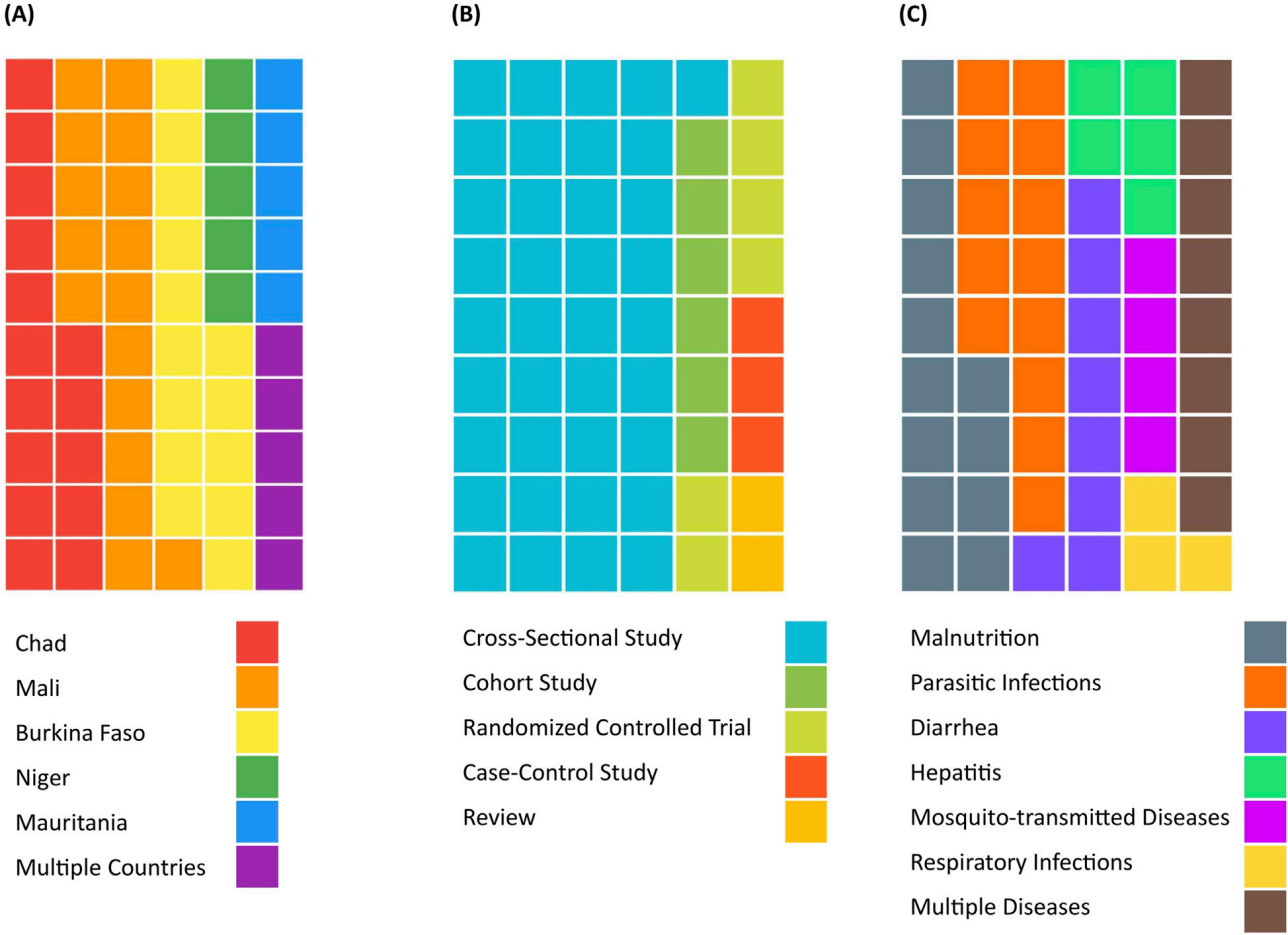
Characteristics of included articles, including the studied country **(A)**, the study design **(B)** and the discussed health outcomes **(C)** (scoping review, Sahel region, 2009–2023).

In total, 54 studies were included in the review. The characteristics of included articles are summarized in Figure 4 and detailed in Supplementary Table S3. The majority of studies reported on WaSH-related health outcomes in three out of the five investigated countries, namely Mali (n = 18), Chad (n = 17) and Burkina Faso (n = 15). Two-thirds of articles reported on cross-sectional studies (n = 37), six on randomized-controlled trials, six on cohort studies, three on case-control studies and two were reviews. The years of publication ranged from 2011 to 2023, with the largest proportion being published in 2023 (n = 10). Almost half of the studies were published after 2020 (n = 25). Of the nine health outcome categories of interest, malnutrition (n = 17), diarrheal diseases (n = 15) and parasitic infections (n = 14) were most commonly reported, followed by hepatitis (n = 7) and respiratory infections (n = 7).

### WaSH-Related Health Outcomes in the Context of Terrorist Activities

One article referred to the security situation in the Sahel region. Lagare et al. reported that Niger has been affected by ongoing Boko Haram attacks since 2015 [21]. These attacks led to about a quarter of a million IDPs in the Diffa Region who resided in refugee camps. The authors described a hepatitis E outbreak in an IDP camp, attributable to the high number of refugees surpassing sanitation and hygiene capacities, as well as straining the fresh water supply. Additionally, the high number of people coming from different regions with different health profiles were mentioned to have potentially supported such an outbreak situation [21].

### Associations of Health Outcomes with WaSH

A broad range of associations between health outcomes and different aspects of WaSH were described across the included articles (Table 1). Improved hygiene, drinking water and sanitation infrastructure were associated with a reduction of most WaSH related health outcomes, whereas exposure to an open water body was strongly linked with an increased risk of parasitic infections [22, 23]. Malnutrition and diarrheal diseases were the most commonly reported health outcomes, usually observed in children under 5 years (Supplementary Table S4) [24–26]. Reported malnutrition spanned different levels of severity from underweight to severe acute malnutrition, stunting and wasting. The most reported parasitic infection was Schistosomiasis.

**TABLE 1:** Reported statistically significant associations of health outcomes with aspects of water, sanitation and hygiene (WaSH), across the 54 included studies^a^ (scoping review, Sahel region, 2009–2023).

	Malnutrition	Diarrheal diseases	Parasitic infections	Hepatitis	Respiratory infections	Mosquito transmitted diseases
Water-Satisfaction	↓					
Improved Sanitation Infrastructure	↓↓	↓↓↓	↓	↓↓	↓	
Improved Hygiene Infrastructure	↓↓	↓	↓	↓	↓	↓
Improved Drinking Water Infrastructure	↓↓↓	↓↓	↓	↓		
Open Defecation	↑					
WaSH-Interventions	↓↓		↓			
Other WaSH-related Health Outcomes	↑ ↑	↑				
Improved School WaSH Infrastructure		↓			↓	
Water Exposure Through Domestic Chores, Swimming or Work			↑↑↑↑	↑		

^a^ The number of arrows represents the number of articles reporting a significant, quantitative association, whereas the direction of the arrow depicts the direction of the association. The positive associations are shown in orange, while the negative associations are blue. The darker the color, the more articles have reported a significant association.

### Vulnerable Groups Affected by WaSH-Related Health Outcomes

Across the included studies, we identified multiple groups of people as particularly vulnerable to inadequate WaSH (Supplementary Table S4). These populations included children under 5 years of age, healthcare and sanitation workers, domestic servants, prisoners, and IDPs [21–31]. Qualitative studies reported a lack of personal protective equipment and multiple WaSH-related health outcomes stemming from fecal and pathogen exposure in healthcare and sanitation workers [27, 28]. Additionally, malaria exposure was high for domestic servants and healthcare workers due to a lack of environmental hygiene and limited protection with bed nets [27, 29]. Furthermore, overcrowding, poor hygienic conditions and inadequate water infrastructure could be linked to a higher infection risk in IDPs and prisoners, including diarrheal diseases, scabies, malnutrition, respiratory tract infections, and hepatitis E [21, 30]. For example, a Hepatitis E outbreak in an IDP camp in Niger affected 38,400 per 100,000 persons compared to an outbreak in an urban environment in Chad the number of people affected was 19 times higher [21, 32]. Out of 100,000 prisoners in Burkina Faso, 23,000 suffered from respiratory complaints [30].

Reported gender-specific vulnerabilities varied depending on the disease (Supplementary Table S4). Parasitic infection and hepatitis E were more strongly associated with women due to a higher exposure to unsafe water during their role in water collection and household activities and due to a greater vulnerability during pregnancy [33–36]. Underweight was more prevalent in male individuals, with men having a 1.5-fold higher risk of being underweight compared to women, possibly associated with a higher exposure to factors contributing to chronic undernutrition [37–39]. However, the authors reported that specific reasons remain unclear and are likely context-specific, potentially involving both sociocultural norms influencing eating practices and physiological factors [39]. Finally, children under the age of five experienced high incidences of diarrhea, with more than 20,000 cases reported over a two-week period [40]. This was strongly associated with malnutrition (OR = 1.25, 95% CI: [0.99; 1.57]) [41], parasitic infections and anemia, reflecting their heightened vulnerability due to their direct environmental exposure through activities such as swimming or playing in contaminated water [31, 34, 41–48].

### Identified Issues, WaSH-Related Needs and Measures

The wide spectrum of reported health outcomes in the Sahel region highlights significant issues that contribute to a substantial burden of disease. The issues reported by authors of included articles were categorized into (i) a lack of WaSH infrastructure, (ii) a lack of knowledge and awareness, (iii) a lack of access to healthcare and (iv) overcrowding (Supplementary Table S4).

Across the included articles, each disease was found to be associated with a lack of WaSH infrastructure by at least one study [28, 30, 31, 39, 40, 44, 49–57], with the exception of anemia. Infrastructure deficiencies concerned a lack of drinking water, insufficient access to handwashing facilities and inadequate access to improved sanitation, which resulted in elevated prevalence of diseases like malnutrition (lower mid-upper arm circumference in children living in a home with unimproved sanitation (adjusted OR = 1.60, 95% CI: [1.11; 2.31] [44]): or parasitic infections (infection associated with tap water (OR = 29.00, 95% CI: [20.89; 38.70]) and unmaintained latrines (OR = 2.37, 95% CI: [0.62; 3.78]) [50]). For diarrhea and hepatitis, the reduction of microorganisms in contaminated water has been reported as a crucial strategy [21]. One article recommended treatment of contaminated water [21] and multiple articles recommended an increased provision of water sources [58–60]. Furthermore, the authors of one article recommended the investigation of possible sources of contamination [61]. Seven articles mentioned the importance of improving sanitation infrastructure, such as latrines and sewage to reduce the risks of cholera, diarrhea and malnutrition [49, 58–60, 62–64]. In contrast, only one article recommended the approach of community-led total sanitation [49].

WaSH-related knowledge and awareness was lacking in study populations and contributed to the development of malnutrition, diarrheal diseases, parasitic infections and hepatitis [34, 41, 42, 45, 61, 65, 66]. Therefore, educational activities targeting the importance of WaSH infrastructure and practices together with knowledge about disease transmission were recommended by 13 articles [32–34, 41–43, 45, 61, 65–69]. Since access to healthcare was limited in most study settings, efforts such as enhanced access to care and regular mass treatment for vulnerable groups were suggested in ten articles, to support individuals affected by malnutrition, parasitic infections as schistosomiasis, malaria, diarrheal diseases, hepatitis and anemia [21, 41–43, 45, 55, 56, 68, 70, 71]. Three articles identified schools as an optimal entry and outreach point for programs tackling the WaSH-related illnesses malnutrition, and parasitic infections [43, 51, 71].

Due to the exceptional circumstances affecting IDPs, one article recommended adapting preventive strategies, such as physical distancing guidelines, to address the challenges of overcrowded camps and limited infrastructure during the COVID-19 pandemic [54]. Generally, articles advised to involve communities in improvement measures by establishing participatory education programs or community-led total sanitation, which cater for individual populations [33, 49]. Additionally, three articles emphasized that diseases, preventive measures, diagnostics or treatment regimes should be tightly monitored with specific indicators to successfully modify interventions and strategies [37, 56, 72].

A lack of appropriate WaSH infrastructure and overcrowding have been identified as factors linked to Boko Haram activities [21]. WaSH infrastructure in an IDP camp could not adequately cover the number of people who were forced to migrate due to the humanitarian crisis, leading to a Hepatitis E outbreak.

## Discussion

This scoping review aimed to summarize the current findings regarding WaSH-related health outcomes in the former G5 Sahel countries linked to terrorist activities, and to identify gaps to improve WaSH-related health in these contexts, by searching the two databases PubMed and Web of Science. We identified only one article that mentioned terrorist activities linked to WaSH-related health outcomes. A total of 54 articles reported WaSH-related health outcomes in the time frames of terrorist insurgency, with the majority of articles being cross-sectional studies situated in Mali, Chad and Burkina Faso and reporting malnutrition. Identified gaps related to poor health outcomes were a lack of WaSH infrastructure, knowledge and awareness, access to healthcare and overcrowding.

### WaSH-Related Health Outcomes in the Context of Terrorist Activities

Terrorist activities were only mentioned by one of the included articles, describing a hepatitis E outbreak in an IDP camp in Niger [21]. The scarcity of literature containing primary data on the association of terrorism with WaSH-related health outcomes highlights the necessity for more research in this highly neglected topic.

However, other articles that did not meet our inclusion criteria because of their use of secondary data provided valuable additional information. A global analysis concluded that with every percentage of increased death caused by terrorism or related violence, a 0.16% increase in DALYs of WaSH-related health outcomes (i.e. diarrheal diseases, schistosomiasis, trachoma and nematode infections) was measurable [73]. Children under five were identified as particularly vulnerable to increasing DALYs, even more so, if they were suffering from malnutrition. This heightened vulnerability stems from interruptions to routine child vaccinations, severe limitations in accessing basic healthcare, and frequent obstacles to the provision of essential resources, including adequate nutrition and safe drinking water [74, 75, 76]. The authors identified functioning WaSH infrastructure as a protective effect of mentioned DALYs. Similarly, high child mortality was associated with the destruction of water and sanitation facilities by terrorist activities in Mali [77]. Such activities are likely to exacerbate the practice of open defecation. Considering the results of our literature review, we conclude that children in areas affected by terrorism might be particularly vulnerable due to limited access to a stable economy, food and healthcare, together with limited access to WaSH infrastructure, especially if they are internally displaced.

Furthermore, recent literature highlights that terrorism in the sub-Saharan region significantly affected the lifetime risk of maternal death (β = 0.001, *p* < 0.01) and the maternal mortality ratio (β = 0.016–0.021, *p* < 0.01) [78]. According to the WHO, maternal mortality ratios in conflict affected settings are five times higher than in stable contexts, reaching 504 maternal deaths per 100,000 live births [79]. One possible underlying factor of increased maternal mortality is that exposure to armed conflicts in sub-Saharan Africa is frequently associated with a decline in the use of essential health services for mothers and young children, such as antenatal care, skilled birth attendance, postnatal care, family planning, and childhood vaccinations [80–82]. Not having access to care can drive women to give birth at home in conditions with limited hygiene. Additionally, they might miss hygiene advice given during antenatal and postnatal care, potentially leading to higher infection risks [83, 84]. In addition, UNICEF reports that children in armed conflicts are more likely to be displaced, face acute malnutrition, and miss critical vaccinations, increasing their susceptibility to disease [85]. Although the articles included in this review never explicitly mentioned reduced access to care due to terrorist activities within their study populations, reduced access to healthcare has been mentioned as an issue in general. These combined effects underline why women and children under five are particularly vulnerable to WaSH-related health outcomes in terrorism-affected regions.

Vertical programs and interventions to monitor and control diseases have also been disrupted by political instability and security challenges. For instance, the dracunculiasis eradication program experienced a massive set back in Mali due to political barriers [86]. Furthermore, implementation challenges, such as the provision of safe WaSH infrastructure prevail with limited access due to insecurity [87]. Ultimately, terrorist activities and related insecurity can hamper governments’ and humanitarian access to populations in need of healthcare or WaSH infrastructure support.

Nevertheless, it is important to note that the state of WASH infrastructure was already subpar prior to the onset of conflicts in the Sahel. Insufficient WaSH facilities were one of the most consistent associations with poor health outcomes in this review [27, 39, 40, 44, 45, 49, 50, 52, 53, 55, 58, 62, 71]. Furthermore, the COVID-19 pandemic has additionally strained already fragile WaSH systems in the Sahel [88, 89]. Lockdowns, supply chain disruptions and reduced humanitarian access delayed maintenance and expansion of facilities. These constraints often forced resources to be reallocated towards immediate pandemic response, leaving structural WaSH issues unresolved and in some areas exacerbated. Terrorist activities may directly affect existing WaSH services through the destruction of facilities and indirectly by causing additional delays in maintenance, limiting supply deliveries or displacing technical staff [90, 91]. In summary, terrorist activities have a direct or indirect impact on the already limited WaSH infrastructure in the Sahel, potentially leading to an increase in DALYs and mortality of WaSH-related diseases. Mothers and children under 5 years of age are particularly vulnerable to these effects. More research investigating direct and indirect effects of terrorist activities on WaSH infrastructure and public health implications is critical to better protect affected populations. To enable research in these complex settings with security concerns, limited accessibility, highly vulnerable populations and restricted resources, interdisciplinary projects with academic and humanitarian institutions are highly recommended.

### Identified Gaps and Measures for the Prevention of WaSH-Related Health Outcomes

Inadequate WaSH infrastructure, knowledge, awareness and inaccessibility to healthcare were the most frequently cited gaps leading to WaSH-related health outcomes. The included articles discussed a range of possible measures to counteract the gaps concerning WaSH-related health outcomes. These included improving WaSH infrastructure, water filtration or Aquatabs, ensuring access to personal hygiene resources by distributing hygiene kits, and enhancing community awareness through education [21, 30, 31, 47, 49, 53, 55, 61]. While physical distancing in crowded areas was mentioned, the implementation in settings affected by war and displacement is often highly challenging [54].

Even though these strategies are important and often applied, some potential barriers, such as local availability of material, need to be overcome.

### Identified Recommendations for the Prevention of WaSH-Related Health Outcomes

Building on the identified gaps and existing measures, we propose the following recommendations to increase the likelihood of long-term solutions. First, solutions and approaches should always involve the affected communities and authorities from the beginning, including the design and implementation of interventions [92]. This methodology will improve cultural acceptance, realistic long-term sustainability and collaboration across different sectors [70]. Second, if possible, materials to build or improve infrastructure should be available or produced locally to guarantee access. Third, if communities are difficult to reach, social and community spaces such as schools [51, 71], mosques, churches or community groups can be used as points of entry. These spaces can serve as hubs for educational programs, distribution of hygiene kits and community-led initiatives. Fourth, when providing educational or behavior change campaigns, we recommend combining them with infrastructure improvements to offer an environment for preventive actions [70]. In conflict-affected contexts, such educational efforts can also be used to strengthen community resilience and reduce the influence of extremist ideologies [93]. Finally, we recommend continuous monitoring and evaluation activities, ideally led by the local communities. A great example of such an approach is the water and sanitation for health facility improvement tool (WASH FIT) that is led and implemented by a committee of community members [94]. This system is now being tested in Mali and Burkina Faso and has already demonstrated great ownership of the communities [95]. Such an approach could also be adapted for schools, detention centers or refugee camps.

To summarize, we recommend a holistic, sustainable, transdisciplinary approach, which combines various measures to improve infrastructure, knowledge and access to healthcare, as well as involves local communities and behavior changing campaigns. However, an optimal solution for this complex situation remains to be developed.

### Strengths and Limitations

This review has some notable strengths. First, it is the first scoping review focusing on WaSH-related health outcomes in the former G5 countries with an emphasis on the overall population. By having this focus, the review provides a comprehensive overview of the health outcomes in the G5 Sahel countries, allowing the identification of vulnerable populations and remaining gaps in the humanitarian and political response with the aim to support humanitarian aid organizations, policymakers and non-governmental organizations (NGOs) in decision-making, and to provide guidance for further action plans. Second, by highlighting the connection between living in insecurity and WaSH-related health outcomes, this review underlines the importance of respecting the human right to clean water and sanitation by all involved conflict parties [96]. Third, we conducted this review in line with the PRISMA standards, allowing reproducibility and transparency. Finally, the literature was screened in a double screening process, which contributes to the quality assurance of the review and reduces potential biases.

Despite its strengths, this review has some limitations. First, to study the relationship between terrorist activities and health outcomes, only articles incorporating data collected after years of commence of insurgencies, were included. Consequently, there is no certainty that terrorist activities solely impacted the reported health outcomes or if they coincided during the same period. To establish a clear association, a meta-analysis with data incorporating safe zones vs. unsafe zones or data from before and after terrorist activities would have to be conducted. However, this review can inform such an analysis in the near future. Second, only diseases associated with WaSH as defined by the WHO were incorporated in this review. Yet, reports from the region suggest that also other health outcomes, such as gender-based violence or mental health are linked to inadequate WaSH [87, 97, 98]. For example, when women and girls are forced to gather water from distant locations they, can be at increased risk of violence. Third, by not including French articles, we might have introduced a language bias and missed relevant studies. In addition, some potentially relevant studies could not be included because their full texts were not accessible, which may have further limited the comprehensiveness of this review. Lastly, a key limitation of this scoping review is the restriction to only two public health databases (PubMed and Web of Science), which may have resulted in the omission of relevant studies. As the review was intended to provide a preliminary overview of health outcomes rather than a comprehensive systematic analysis, the findings should be interpreted within this limited scope.

### Conclusions

This review documented the scarcity of data about the impact of terrorist activities on WaSH-related health outcomes in the former G5 Sahel region, with only one study linking Hepatitis E with the humanitarian crisis caused by terrorism. However, we identified an array of WaSH-related health outcomes, such as malnutrition, diarrheal and parasitic diseases that are highly prevalent in the Sahel, these diseases disproportionately affect vulnerable populations and are likely to be linked to terrorist activities. Terrorism further limits access to healthcare and WaSH infrastructure, particularly increasing the risk of WaSH-related mortality and DALYs in children under five. Women are similarly vulnerable, as conflict often disrupts access to maternal and reproductive health services, which may heighten risks of infection and maternal mortality in settings where hygiene and safe delivery are not guaranteed. Additionally, this review highlights the difficulties caused by the security situation and political instabilities for governments and humanitarian actors to support impacted populations with material, infrastructure and interventions. Terrorist activities not only damage or weaken existing WaSH infrastructure, but also restrict governmental and humanitarian access and delay essential services. These effects compound already fragile systems, making sustainable prevention and response even more challenging.

To address the identified gaps in infrastructure, access to healthcare, knowledge and awareness, for the improvement of WaSH-related health outcomes in the region, targeted, evidence-based and holistic interventions are needed that involve local communities and experts from the onset. Such participatory and transdisciplinary approaches are crucial to ensure acceptance, resilience, and long-term sustainability. At the same time, epidemiological studies to capture both, direct and indirect effects of terrorism on WaSH and health are required to provide stronger guidance for policymakers and humanitarian action. These efforts should be targeted by joint collaborations of researchers, local governments and humanitarian actors.
