# Supplementary material for: Exploring Associations Between WaSH-Related Health Outcomes and Terrorist Activities in the Sahel: A Scoping Review
Source: Public Health Rev. 2025 Oct 23;46:1608490. doi: 10.3389/phrs.2025.1608490 (PMC12588887; doi:10.3389/phrs.2025.1608490)
Supplement: Supplementary file 1 [file DataSheet1.pdf]

Supplementary Material

Supplementary Table 1: Databases and search strategy combining G5 countries with WaSH-related terms

Database	Query [1]
**PubMed**	("Mauritania"[Title/Abstract] AND 2009/01/01:2023/12/22[Date - Publication]) OR ("burkina faso"[Title/Abstract] AND 2015/01/01:2023/12/22[Date - Publication]) OR ("Niger"[Title/Abstract] AND 2015/01/01:2023/12/22[Date - Publication]) OR ("Chad"[Title/Abstract] AND 2015/01/01:2023/12/22[Date - Publication]) OR ("Mali"[Title/Abstract] AND 2012/01/01:2023/12/22[Date - Publication])
	AND
	("Water"[MeSH Terms] OR "Sanitation"[MeSH Terms] OR "Hygiene"[MeSH Terms] OR "Water"[Title/Abstract] OR "Sanitation"[Title/Abstract] OR "Hygiene"[Title/Abstract] OR "Soap"[Title/Abstract] OR "Latrine"[Title/Abstract] OR "Wash"[Title/Abstract] OR "hand washing"[Title/Abstract])
	NOT
	("aspergillus niger"[MeSH Terms] OR ("aspergillus"[All Fields] AND "Niger"[All Fields]) OR "aspergillus niger"[All Fields])
**Web of Science**	(((TI=(Chad)) OR AB=(Chad)) AND DOP=(2015-01-01/2023-12-22)) OR (((TI=(Burkina Faso)) OR AB=(Burkina Faso)) AND DOP=(2015-01-01/2023-12-22)) OR (((TI=(Mali)) OR AB=(Mali)) AND DOP=(2012-01-01/2023-12-22)) OR (((TI=(Niger)) OR AB=(Niger)) AND DOP=(2015-01-01/2023-12-22)) OR (((TI=(Mauritania)) OR AB=(Mauritania)) AND DOP=(2009-01-01/2023-12-22))
	AND
	(((((((((((((AB=(water)) OR AB=(sanitation)) OR AB=(hygiene)) OR AB=(soap)) OR AB=(wash)) OR AB=(latrine)) OR AB=(hand*washing)) OR TI=(water)) OR TI=(sanitation)) OR TI=(hygiene)) OR TI=(soap)) OR TI=(wash)) OR TI=(latrine)) OR TI=(hand*washing)))
	AND NOT
	(TI=("Aspergillus niger")) OR AB=("Aspergillus niger")

[1] *We initially tested search terms related to terrorism and armed conflict. However, including such terms considerably reduced the number of relevant articles retrieved, excluding many studies addressing the indirect effects of insecurity on WaSH and health outcomes. Therefore, we opted for a broader, more neutral search strategy to ensure that both direct and indirect impacts of regional instability could be captured. This decision aligns with the exploratory nature of a scoping review.*

Supplementary Table 2: Inclusion and exclusion criteria

	**Inclusion Criteria**	**Exclusion Criteria**
**Study Characteristics**	- Peer-reviewed scientific literature with primary data<br>- Scientific reviews	- Study protocols<br>- News<br>- Methods Papers<br>- Editorials<br>- Comments<br>- Plaidoyers / Funding calls from non-governmental organizations (NGOs)
**Population**	- People residing in countries formerly known as the G5 Nations (Burkina Faso, Chad, Niger, Mali, Mauritania)	
**Outcome**	- Diseases associated with inadequate Water, Sanitation and Hygiene as listed by the WHO (1), see Figure 2	
**Time Frame**	- Study starting from the respective years of terrorist activities (if two countries featured in one paper, inclusion according to the country with longer-standing terrorist activities)	

Supplementary Table 3: Characteristics of the included articles

Publication	Year of Publication	Study Period	Countries	Study Type	Health Outcomes
Altmann et al. (40)	2018	2015-2016	Chad	Cluster-Randomized Controlled Trial	Malnutrition
Bountogo et al. (38)	2021	2018-2019	Burkina Faso	Cross-Sectional Study	Malnutrition
Darling et al. (32)	2020	2015-2017	Burkina Faso	Cross-Sectional Study	Malnutrition
Diendéré et al. (24)	2022	2017	Burkina Faso	Cross-Sectional Study	Malnutrition, Diarrheal Diseases, Respiratory Infections, Scabies
Dodos et al. (57)	2018	2017	Chad	Case-Control Study	Malnutrition, Diarrheal Diseases
Erismann, Knoblauch, et al. (45)	2017	2015	Burkina Faso	Cross-Sectional Study	Malnutrition
Fagbamigbe, Kandala and Uthman (60)	2020	2012-2019	Chad<br>Mali<br>Niger	Cross-Sectional Study	Malnutrition
Gelli et al. (41)	2019	2017	Burkina Faso	Cross-Sectional Study	Malnutrition
Huybregts et al. (36)	2019	2015-2017	Mali	Cluster-Randomized Controlled Trial	Malnutrition, Anemia
Ibro et al. (39)	2022	2016-2017	Niger	Cross-Sectional Study	Malnutrition
Kangas et al. (42)	2023	2020-2021	Mali	Cohort Study	Malnutrition
Marshak, Young, and Naumova (91)	2023	2018-2020	Chad	Cohort Study	Malnutrition
Marshak et al. (31)	2021	2015-2017	Chad	Cluster-Randomized Controlled Trial	Malnutrition
Pinchoff, Turner, and Grace (35)	2021	2017	Burkina Faso	Cross-Sectional Study	Malnutrition, Diarrheal Diseases
Prado et al. (92)	2019	2018	Burkina Faso	Cohort Study	Malnutrition
Ricci et al. (25)	2019	2000-2015	Burkina Faso<br>Chad<br>Mali<br>Mauritania	Cross-Sectional Study	Malnutrition

Publication	Year of Publication	Study Period	Countries	Study Type	Health Outcomes
			Niger		
Sié et al. (33)	2022	2019-2020	Burkina Faso Mali Niger	Cross-Sectional Study	Malnutrition
Anderson et al. (21)	2023	2022	Niger	Cross-Sectional Study	Diarrheal Diseases
Berendes, Fagerli, et al. (56)	2023	2015-2018	Mali	Cross-Sectional Study	Diarrheal Diseases
Berendes, Omore, et al. (93)	2023	2015-2018	Mali	Case-Control Study	Diarrheal Diseases
Chard et al. (53)	2018	2014	Mali	Cluster-Randomized Controlled Trial	Diarrheal Diseases
Garn et al. (51)	2017	2013-2014	Mali	Cohort Study	Diarrheal Diseases, Respiratory Infections
Graveleau et al. (43)	2021	2018	Niger	Cross-Sectional Study	Diarrheal Diseases
Hirai et al. (34)	2017	2010-2014	Mali Mauritania	Cross-Sectional Study	Diarrheal Diseases
Philippe et al. (22)	2022	2019	Burkina Faso	Cross-Sectional Study	Diarrheal Diseases, Hepatitis A, Respiratory Infections, Leptospirosis
Robert et al. (58)	2021	2017	Burkina Faso	Cross-Sectional Study	Diarrheal Diseases
Toure et al. (54)	2019	2018	Mali	Cross-Sectional Study	Diarrheal Diseases, Parasitic Infections (Amoebic dysentery), Hepatitis A
Traoré et al. (55)	2013	2009	Mauritania	Cross-Sectional Study	Diarrheal Diseases
Trinies et al. (52)	2016	2013-2014	Mali	Cohort Study	Diarrheal Diseases, Respiratory Infections
Agniwo et al. (63)	2023	2021	Mali	Cross-Sectional Study	Parasitic Infections (Schistosomiasis)
Cisse et al. (27)	2021	2020	Burkina Faso	Cross-Sectional Study	Parasitic Infections (Schistosomiasis)
Gbalégba et al. (28)	2017	2014-2015	Mauritania	Cross-Sectional Study	Parasitic Infections (Schistosomiasis)
Lalaye, De Bruijn, and De Jong (29)	2019	2015-2016	Chad	Cross-Sectional Study	Parasitic Infections (Schistosomiasis)
Moser et al. (61)	2022	2019	Chad	Cross-Sectional Study	Parasitic Infections (Schistosomiasis)

Publication	Year of Publication	Study Period	Countries	Study Type	Health Outcomes
Willemann Stecher et al. (94)	2017	2017	Mali	Randomized-Controlled Trial	Parasitic Infections (Schistosomiasis)
Erismann et al. (37)	2016	2015	Burkina Faso	Cross-Sectional Study	Parasitic Infections (Intestinal Protozoa)
Erismann, Diagbouga, et al. (64)	2017	2015	Burkina Faso	Cluster-Randomized Controlled Trial	Parasitic Infections (Intestinal Protozoa)
Fofana et al. (95)	2019	2016	Mali	Cross-Sectional Study	Parasitic Infections (Schistosomiasis, Giardiasis)
Galan-Puchades (59)	2020	2020	Chad	Review	Parasitic Infections (Dracunculiasis)
Guagliardo et al. (30)	2022	2019	Chad	Cross-Sectional Study	Parasitic Infections (Dracunculiasis)
Liu et al. (62)	2021	2013-2017	Chad	Case-Control Study	Parasitic Infections (Dracunculiasis)
Nadia et al. (44)	2023	*NA*	Chad	Cross-Sectional Study	Parasitic Infections (Geohelminthiasis)
Dimeglio et al. (46)	2019	2013-2016	Burkina Faso	Cross-Sectional Study	Hepatitis E
Lagare et al. (20)	2018	2017	Niger	Cross-Sectional Study	Hepatitis E
Lenglet et al. (96)	2020	2016-2017	Chad	Cross-Sectional Study	Hepatitis E
Spina et al. (26)	2017	2016	Chad	Cross-Sectional Study	Hepatitis E
Vernier et al. (47)	2018	2017	Chad	Cross-Sectional Study	Hepatitis E
Ag Ahmed et al. (48)	2021	2020	Mali	Cross-Sectional Study	Respiratory Infections (COVID-19)
Cissoko et al. (97)	2023	2020	Mali	Cross-Sectional Study	Respiratory Infections (COVID-19)
Safiri et al. (49)	2023	2019	Chad	Cross-Sectional Study	Respiratory Infections
El Moustapha et al. (50)	2023	2015-2020	Mauritania	Cohort Study	Mosquito Transmitted Diseases (Malaria)
Fiorentino et al. (23)	2023	2021	Mali	Cross-Sectional Study	Mosquito Transmitted Diseases (Malaria)
Lekweiry et al. (65)	2011	2009-2010	Mauritania	Cross-Sectional Study	Mosquito Transmitted Diseases (Malaria)
Gerken et al. (98)	2022	1999-2021	Mali Mauritania Niger	Review	Mosquito Transmitted Diseases (Rift Valley Fever)

Supplementary Table 4: Health Outcome's association with WaSH in the former G5 Sahel member states with epidemiological indicators (i.e. prevalence, incidence and mortality), reported associations with WaSH and recommended preventive measures

Health Outcome	Diagnosis	Population	Prevalence per 100,000[2]	Association with WaSH	Recommended Preventive Measures
**Malnutrition**	Underweight	Children (1-59 months) *WAZ[3] ≤ 2*	19,000 (33)	Increased WAZ (+0.27, 95% CI[4]: [0.09; 0.46]) in children accessing improved latrines (33)	*NA*
		Adolescents (10-19 years) *BMI[5] z-score ≤ -2*	15,100 (32)	*NA*	NA
		Prisoners *BMI < 18.5 kg/m2*	8,000 (24)	Prisoners lacking adequate hygiene (24)	Improved personal hygiene and environmental conditions (24)<br>Reduction of overcrowded conditions (24)
	Moderate Acute Malnutrition	Children (1-59 months) *MUAC[6] = 11.5 - 12.5 cm*	9,800 (33)	*NA*	Nutritional Interventions (33)
		Children (6-23 months) *MUAC < 13.5 cm*	34,000 (35)	Association of increased Water Requirement Satisfaction Index with lower odds of malnutrition (OR[7] = 0.971, 95% CI: [0.942; 1.00]) (35)<br>Association of diarrhea (other WaSH-related health outcome) with higher odds of malnutrition (OR = 1.249, 95% CI: [0.993; 1.570]) (35)	Maternal education (35)<br>Access to health care (35)

[2] If no time frame defined, prevalence at time of study
[3] WAZ: Weight-for-age z-score
[4] CI: Confidence Interval
[5] BMI: Body Mass Index
[6] MUAC: Middle upper arm circumference
[7] OR: Odds ratio

Health Outcome	Diagnosis	Population	Prevalence per 100,000[2]	Association with WaSH	Recommended Preventive Measures
		Children (6-59 months)<br>*MUAC < 12.5 cm* (38)<br>*MUAC = 11.5-12.4 cm* (39)	2,900 (38)<br>7,600 (39)	Lower MUAC in children living in a home with unimproved sanitation (aOR[8] = 1.60, 95% CI: [1.11; 2.3]) or practicing open defecation (aOR = 1.36, 95% CI: [0.94; 1.98]) compared to accessing improved sanitation (38)<br>Lower MUAC in children living in a home using a dug well (aOR = 1.12, 95% CI: [0.91; 1.37]) compared to accessing a borehole (38)<br>Association of malnutrition with lacking access to improved sanitation (39)	Access to improved Sanitation (38,57)<br>Improved hygiene of guardians (57)<br>Comprehensive approach including nutrition, education, improved sanitation, hygiene and health care (39)
		Mothers<br>*MUAC = 25-22 cm*	24,000 (35)	*NA*	Maternal education (35)<br>Access to health care (35)
	Malnutrition	School children<br>*(Child stunted, thin underweight or overweight)*	37,100 (45)	Correlation of malnutrition with other WaSH-related health outcomes (parasitic infection (aOR = 1.87, 95% CI: [1.02; 3.43]), anemia (aOR = 2.52, 95% CI: [1.25; 5.08])) (45)	School-based WaSH interventions (45)
	Severe acute Malnutrition	Children (1-59 months)<br>*MUAC < 11.5 cm*	1,000 (33)	*NA*	Nutritional Interventions (33)
		Children (6-23 months)<br>*MUAC < 11.5 cm*	2,700 (36)	*NA*	Nutritional Interventions (36)<br>Education (36)
		Children (6-59 months)<br>*MUAC < 11.5 cm*	300 (38)<br>3,700 (39)	Lower MUAC in children living in a home with unimproved sanitation (aOR = 1.60, 95% CI: [1.11; 2.31]) or practicing open defecation (aOR = 1.36, 95% CI: [0.94; 1.98]) compared to accessing improved sanitation (38)	Comprehensive approach including nutrition, education, improved sanitation, hygiene and health care (39)

[8] aOR: Adjusted odds ratio

Health Outcome	Diagnosis	Population	Prevalence per 100,000[2]	Association with WaSH	Recommended Preventive Measures
				Lower MUAC in children living in a home using a dug well (aOR = 1.12, 95% CI: [0.91; 1.37]) compared to accessing a borehole (38)<br><br>Association of malnutrition with lacking access to improved sanitation (39)	
		Mothers<br>*MUAC ≤ 22 cm*	2,000 (35)	*NA*	Maternal education (35)<br><br>Access to health care (35)
	Wasting<br>*WHZ*[9] *< -2*	Children (0-59 months)	23,070 (Mauritania) (25)<br><br>25,400 (Mali) (25)<br><br>31,080 (Burkina Faso) (25)<br><br>39,940 (Niger) (25)<br><br>30,620 (Chad) (25)	1.87% (95% CI: [0.38; 3.36]) decrease in underweight with 10% increase in access to water (25)	Increased access to water (25)
		Children (6-59 months)	6,000-17,000 (41)<br><br>12,000 (91)<br><br>13,700 (31)	Seasonality of wasting with 2 peaks annually (May and October) related to the beginning and end of rainy season (91)<br><br>Decrease from 13.7% (95% CI: [10; 18]) to 11.5% (95% CI: [6; 17] in wasting in children with nutrition intervention containing WaSH (31)	Education (41)<br><br>WaSH Programs (41)

[9] WHZ: Weight-for-height z-score

Health Outcome	Diagnosis	Population	Prevalence per 100,000[2]	Association with WaSH	Recommended Preventive Measures
	Severe Wasting *WHZ < -3*	Children (6-59 months) in health centers	75,800 (40)<br>4,850 (Mali) (60)<br>4,350 (Chad) (60)<br>6100 (Niger) (60)<br>1,400 (91)	Increased rate of recovery (+ 10.5%, 95% CI: [6.7; 19.8]) and higher weight gain (+3g/d, 95% CI: [0.6; 5.4]) with WaSH intervention (40)<br>Higher odds in rural areas, association with source of drinking water (60)	WaSH packages for households (40)<br>Nutritional interventions (60)<br>Education (60)
	Stunting *HAZ*[10] *< -2*	Children (6-59 months)	19,000-26,000 (41)	Association of higher HAZ score with primarily accessing a borehole for drinking water (multivariate linear regression coefficient = 0.14, p[11] = 0.004) and the child being visually clean (multivariate linear regression coefficient = 0.15, p = 0.002) (41)	Education (41)<br>WaSH Programs (41)
		School children (8-14 years)	29,400 (45)	Correlation of malnutrition with other WaSH-related health outcomes (parasitic infection (aOR = 1.87, 95% CI: [1.02; 3.43]), anemia (aOR = 2.52, 95% CI: [1.25; 5.08])) (45)	School-based WaSH interventions (45)
		Adolescents (10-19 years)	14,500 (32)	*NA*	*NA*
	Severe Stunting *HAZ < -3*	Children (6-59 months) in health centers	61,000 (40)	Increased rate of recovery (+ 10.5%, 95% CI: [6.7; 19.8]) and higher weight gain (+3g/d, 95% CI: [0.6; 5.4]) with WaSH intervention (40)	WaSH packages for households (40)

[10] HAZ: Height-for-age z-score
[11] p: P-value

Health Outcome	Diagnosis	Population	Prevalence per 100,000[2]	Association with WaSH	Recommended Preventive Measures
	Malnutrition Relapse<br>*MUAC < 12.5cm*	Children recovered from malnutrition	23,600, 6 months (42)	Association of relapse with lacking improved source of water (aHR[12] = 1.93, 95% CI: [1.16; 3.20]) (42)	Improved access to safe drinking water (42)
**Diarrheal Diseases**	Diarrhea	Children (0-59 months)	<10,000, current<br>>20,000, 2 weeks (34)	Association of improved sanitation (PR = 0.86, 95% CI: [0.83; 0.89]), piped water as source for drinking water (PR = 0.88, 95% CI: [0.84; 0.93]) and proximity to water source (less than 30 minutes) (PR = 0.93, 95% CI: [0.91; 0.95]) with diarrhea prevalence (34)	Improved water and sanitation infrastructure (56)<br>Promotion of handwashing with soap (34)<br>Access to improved sanitation facilities (57)<br>Improved hygiene of guardians (57)
		Children (6-23 months)	33,000, 2 weeks (35)	Association of diarrhea with higher odds of malnutrition (other WaSH-related health outcome) (OR = 1.249, 95% CI: [0.993; 1.570]) (35)	Maternal Education (35)<br>Access to health care (35)
		School children	*NA*	Lower antibody evidence of enteric diseases in pupils attending schools implementing WaSH program (53)<br>Association of improved sanitation with lower odds of diarrhea (OR = 0.68, 95% CI: [0.5; 0.93]) (51)<br>Lower risk of suffering from diarrhea in pupils attending schools that fulfill WaSH targets (OR = 0.65, 95% CI: [0.47; 0.92]) (51); (OR = 0.71, 95% CI: 0.6; 0.85) (52)	Improved access to safe drinking water, soap and improved sanitation infrastructure (51–53)
		Health care workers	*NA*	Health care workers exposed to pathogens due to inadequate sanitation (21)	Improved access to soap and water (21)

[12] aHR: Adjusted hazard ratio

Health Outcome	Diagnosis	Population	Prevalence per 100,000[2]	Association with WaSH	Recommended Preventive Measures
		Sanitation workers	*NA*	Diarrhea reported in workers responsible for sanitation (22)	Treatment of pits with kerosene to eliminate pathogens (22)<br>Protective equipment for workers (22)
		Prisoners with digestive complaints	4,200 (24)	Prisoners lacking adequate personal hygiene (24)	Improved personal hygiene and environmental conditions (24)<br>Reduction of overcrowded conditions (24)
		Individuals consulting healthcare	27,000 (58)<br>12,800 (55)	Health center closer to surface water, contaminated by E. coli, reported more diarrhea; Deterioration during rainy season (58)<br>Living at large distance to drinking water points as risk factor for diarrhea (58)<br>Negative correlation between unimproved water infrastructure and diarrhea (55)	Education (58)<br>Increase awareness (58)<br>Access to improved infrastructure (55)
		Residents	55,000-61,000 (54)	Residents who consumed investigated water with high bacterial content often suffered from diarrhea (54)	Education (54)
	Cholera	Residents	1,490 (43)	Access to full sanitation and safe water decreased odds to develop cholera (OR = 0.13, 95% CI: [0.06; 0.26]) (43)	Improved access to WaSH (43)
		Sanitation workers	*NA*	Cholera reported in workers responsible for sanitation (22)	Treatment of pits with kerosene to eliminate pathogens (22)<br>Protective equipment for workers (22)
	Typhoid Fever	Residents	38,000-52,000 (54)	Residents who consumed investigated water with high bacterial content often suffered from typhoid fever (54)	Education (54)
**Parasitic Infections**	Schistosomiasis (*S. haematobium*)	Children (1-14 years)	24,900 (29)	Higher prevalence in males (73%) due to higher exposure through naked water behavior (29)	Increase awareness in parents (29)

Health Outcome	Diagnosis	Population	Prevalence per 100,000[2]	Association with WaSH	Recommended Preventive Measures
		School children	4,000 (28)<br>50,200 (63)<br>3,900 (37)	Reduced odds during dry season (aOR = 0.56, 95% CI: [0.35; 0.89]) (28)<br>Higher odds in males due to swimming (aOR = 1.78, 95% CI: [1.13; 2.80]) (28)<br>Children who gathered water from river, swam in it and regularly urinated in it were more affected (63)<br>Lower odds for S. haematobium infection in children living in household with access to soap (37)<br>Higher odds for S. haematobium infection in children carrying out domestic chores in water (37)	Education (28,37)<br>Improved access to safe WaSH (28,37)<br>Routine large-scale treatment (63)<br>Deworming programs in schools (37,64)
		Residents	39,200 (61)<br>79,800 (94)	Variations across villages associated with proximity to water locations, utilized from swimming and washing (61)<br>Higher prevalence in men and communities relying on fishing for income (94)	Education (61)<br>Improved access to diagnostics and treatment (61)
	Schistosomiasis (*S. mansoni*)	Children (under 6 years)	81,100 (27)	Higher odds in girls (aOR = 2.9, 95% CI: [1.3; 6.1]), due to accompaniment of mother to carry out domestic activities at pond (27)<br>Higher odds of infection when living proximity to pond (aOR = 3.0, 95% CI: [1.0; 8.6]) or going in pond (aOR = 5.0, 95% CI: [1.7; 14.3]) (27)	Participatory education (27)<br>Minimize exposure to pond water (27)
		School children (8-14 years)	300 (37)	*NA*	Education (37)<br>Improved access to safe WaSH (37)

Health Outcome	Diagnosis	Population	Prevalence per 100,000[2]	Association with WaSH	Recommended Preventive Measures
					Deworming programs in schools (37,64)
		Residents	41,000 (95)	*NA*	*NA*
	Dracunculiasis	Residents	17 cases, in 2018 (59)<br>48 cases, in 2019 (30)	Reduction of incidence by 99.99% through eradication program, including filtering and treatment of drinking water (59)<br>Consumption of unfiltered water by all cases (30)<br>More women affected (59%), likely due to collection of water (30)	Education (59,62)<br>Filtering water for dirking (30,59)<br>Water treatment with larvicides (59)
	Geohelminthiasis	School children (5-15 years)	16,520 (44)	Higher prevalence in age group 9-13 (OR = 1.997, 95% CI: [1.085; 3.677]), likely due to less hygienic behavior and playing in soil (44)<br>Infection associated with tap water (OR = 29, 95% CI: [20.89; 38.70]) and unmaintained latrines (OR = 2.37, 95% CI: [0.62; 3.78]) (44)	Improved hygiene (44)
	Amoebic Dysentery	Residents	31,000-39,000 (54)	Residents who consumed investigated water with high bacterial content often suffered from amoebic dysentery (54)	Education (54)
	Giardiasis	Residents	38,000 (95)	*NA*	*NA*
	Intestinal Protozoa	School children	80,000 (37)<br>88,600 (64)	Reduction of intestinal protozoa through WaSH interventions, including hand washing facilities, improved sanitation and water supply (OR = 0.2, 95% CI: [0.1; 0.5]) (64)	Education (37)<br>Improved access to safe WaSH (37)<br>Deworming programs in schools (37,64)
**Hepatitis**	Hepatitis A	Sanitation workers	*NA*	Hepatitis A reported in workers responsible for sanitation (22)	Treatment of pits with kerosene to eliminate pathogens (22)<br>Protective equipment for workers (22)
		Residents	16,000-29,000 (54)	Residents who consumed investigated water with high bacterial content often suffered from hepatitis A (54)	Source investigation (54)<br>Education (54)

Health Outcome	Diagnosis	Population	Prevalence per 100,000[2]	Association with WaSH	Recommended Preventive Measures
	Hepatitis E	Suspected patients with acute febrile jaundice	2,600 (46)<br>38,400 (20)<br>13,000 (96)<br>2,000 (26)	Higher prevalence in arid norther with more limited supply of drinking water and sanitation services (OR = 2.8, 95% CI: [1.58; 4.97]) (46)<br>Higher prevalence in females (58.6%), due to responsibilities related to collection and management of water (20)<br>Higher median exposure to unsafe water in hepatitis cases (96)<br>Possible link of area of residence, water and sanitation and HEV transmission (26)	Access to improved infrastructure (26,46,96)<br>Aquatabs to decontaminate water (20)<br>Improved access to medical care (20)<br>Handwashing with soap (96)<br>Improved personal hygiene (26)<br>Education (26)
		Residents	7,700, past or recent infection (47)	Association of sharing sanitation infrastructure with other households (PR = 1.72, 95% CI: [1.08; 2.73]) and not using soap for handwashing (PR = 1.85, 95% CI: [1.30; 2.63]) with recent HEV infection (47)	Improved personal hygiene and access to improved infrastructure and soap (47)
**Respiratory Infections**	Respiratory Symptoms	School children	NA	Lower risk of suffering from respiratory symptoms in pupils attending schools that fulfill WaSH targets (aOR = 0.75, 95% CI: [0.56; 1.00] (51), 95% CI: [0.65; 0.86] (52))	Access to water supply, handwashing facilities and sanitation infrastructure (51,52)
		Prisoners with health complaint	23,000 (24)	Prisoners lacking adequate personal hygiene (24)	Improved personal hygiene and environmental conditions (24)<br>Reduction of overcrowded conditions (24)
		Sanitation workers	*NA*	Respiratory symptoms reported in workers responsible for sanitation (22)	Treatment of pits with kerosene to eliminate pathogens (22)<br>Protective equipment for workers (22)

Health Outcome	Diagnosis	Population	Prevalence per 100,000[2]	Association with WaSH	Recommended Preventive Measures
	COVID-19	IDPs	*NA*	Limited number of water points and toilets and sharing of facilities by multiple households as a challenge for implementation of physical distancing in refugee camps (48)	Improvement of overcrowded conditions and infrastructure (48)
		Residents	16,400, estimated (97)	*NA*	Enhance participation of health care workers (97)<br><br>Increase awareness of prevention strategies (97)
	Lower Respiratory Infections	Residents	12,208, 1 year (49)	Access to improved sanitation (aOR = 0.72, 95% CI: [0.64; 0.97]) and handwashing facilities as protective factors (49)	Improvement of health care access and infrastructure (49)
**Mosquito Transmitted Diseases**	Malaria	Febrile children	16,500 (50)<br><br>34,900 (65)	Positive correlation between malaria and rainfall ($r^{13} = 0.85$, $p = 0.031$) (50)<br><br>Reduction in annual prevalence (-27.1% in 5 years, $p < 0.05$) of malaria linked to improvements in network to supply water (50)	Reinforcement of diagnostic, therapeutic and preventive measures (50)<br><br>Surveillance to monitor occurrence (65)
		Young domestic servants	*NA*	Young domestic servants with limited access to water and soap, living in humid conditions reporting malaria (23)	*NA*
		Residents	2,100 (50)	Positive correlation between malaria and rainfall ($r = 0.85$, $p = 0.031$) (50)<br><br>Reduction in annual prevalence (-27.1% in 5 years, $p < 0.05$) of malaria linked to improvements in network to supply water (50)	Reinforcement of diagnostic, therapeutic and preventive measures (50)
	Rift Valley Fever	Residents	Average of 5.8 outbreaks per year (98)	Residing or working near water sources, where mosquitos develop, associated with higher exposure to virus (98)	*NA*

[13] r: Pearson correlation coefficient

Health Outcome	Diagnosis	Population	Prevalence per 100,000[2]	Association with WaSH	Recommended Preventive Measures
**Anemia**	Anemia	Children (6-23 months)	83,000 (36)	*NA*	Enhanced nutrition and health care (36)
**Leptospirosis**	Leptospirosis	Sanitation workers	*NA*	Leptospirosis reported in workers responsible for sanitation (22)	Treatment of pits with kerosene to eliminate pathogens (22)<br><br>Protective equipment for workers (22)
**Scabies**	Scabies	Prisoners with health complaint	1,850 (24)	Prisoners lacking adequate personal hygiene (24)	Improved personal hygiene and environmental conditions (24)<br><br>Reduction of overcrowded conditions (24)

1
